# Metastatic Mucinous Adenocarcinoma of the Cervix Presenting as Acute Hepatitis

**DOI:** 10.7759/cureus.6620

**Published:** 2020-01-10

**Authors:** Shanker Kundumadam, Hansini Laharwani, Shafeek Azhikkalayil, Gokul Das

**Affiliations:** 1 Internal Medicine, Wayne State University School of Medicine, Detroit, USA; 2 Pathology, University of Mississippi Medical Center (UMMC), Jackson, USA; 3 Internal Medicine, Shyam Shah Medical College, Bhopal, IND; 4 Pediatrics, Postgraduate Institute of Medical Education and Research, Chandigarh, IND

**Keywords:** cervical cancer, acute hepatitis, metastatic cervical cancer

## Abstract

Cervical cancer is well known to cause metastasis in liver. Not usually they present in a pattern of acute hepatitis with the liver enzymes trending above the range of thousands. Here we present an interesting case of a patient who presented as acute hepatitis in the setting of metastatic cervical cancer to the liver. She presented with elevated liver enzymes above thousands. Her imaging studies revealed new metastatic lesions in the liver. She was subsequently started on chemotherapy but unfortunately, two months later developed septic shock and expired. To our knowledge this is the only case describing a malignancy with metastasis to the liver presenting as an acute hepatitis picture. All the other cases that have been described in the literature presented with acute liver failure.

## Introduction

Cervical cancer is well known to cause metastasis in liver [1]. Not usually they present in a pattern of acute hepatitis with the liver enzymes trending above the range of thousands. Usually the causes for elevated liver enzymes in that high range are viral hepatitis, ischemic hepatitis, drug or toxin-induced hepatitis [2]. Here we present an interesting case of a patient who presented with acute hepatitis in the setting of metastatic cervical cancer to the liver. This case is interesting as to our knowledge there are not a lot of cases of metastatic cervical cancer in literature which presented as an acute hepatitis, challenging the diagnostic thought process because of the other possible differentials as mentioned above.

## Case presentation

Our patient is a 47-year-old female who has been diagnosed with undifferentiated mucinous cervical adenocarcinoma a year and a half ago and had hysterectomy with salpingo-oophorectomy along with chemoradiation. She presented to the hospital with complaints of fatigue, nausea and reduced appetite for a week. Her examination revealed icterus and mild right upper quadrant tenderness but no ascites. She had no petechial rash and had no features of encephalopathy. Her initial workup revealed elevated liver enzymes with alanine transaminase (ALT) of 1891, aspartate transaminase (AST) of 600 and alkaline phosphatase (ALP) of 178. She had a total bilirubin level of 2.1 with the direct bilirubin of 1.9. Her electrolyte panel, renal parameters, and hemogram were normal.

**Figure 1:**
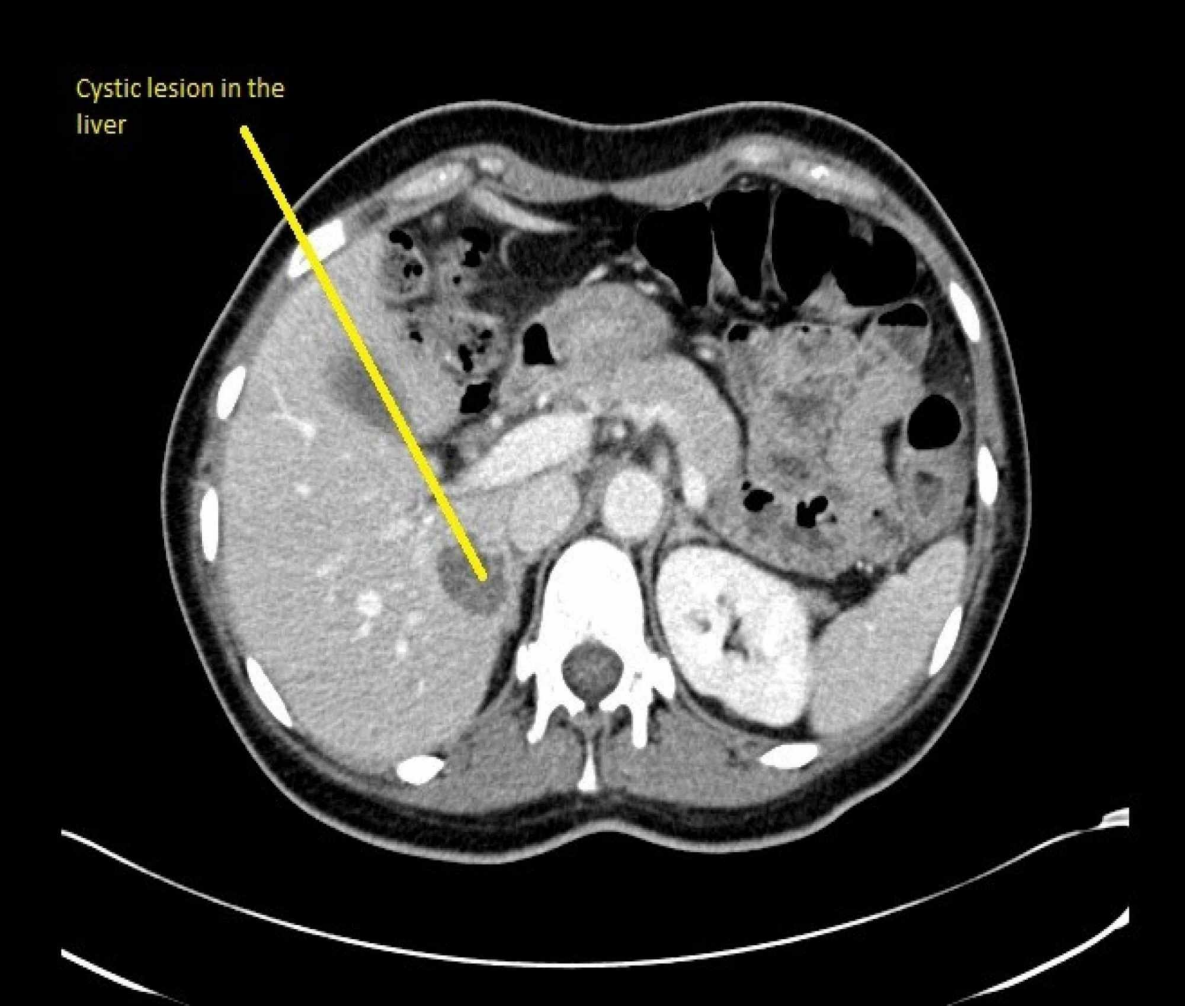
Cystic lesion in the liver suspicious for metastatic lesion

**Figure 2:**
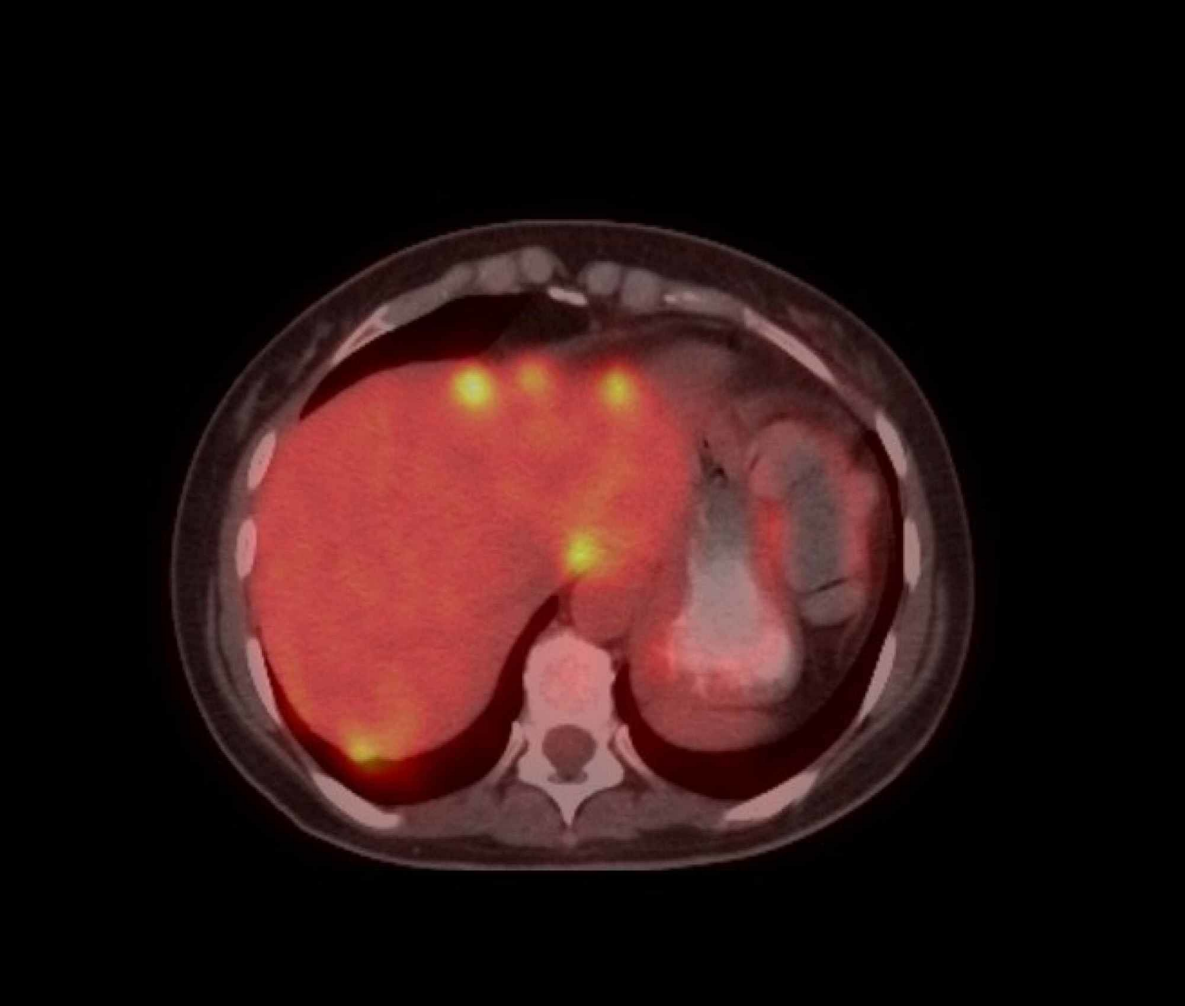
Positron emission tomography (PET) scan done during current presentation showing new metastatic foci in the liver with hyper metabolic activity

**Figure 3:**
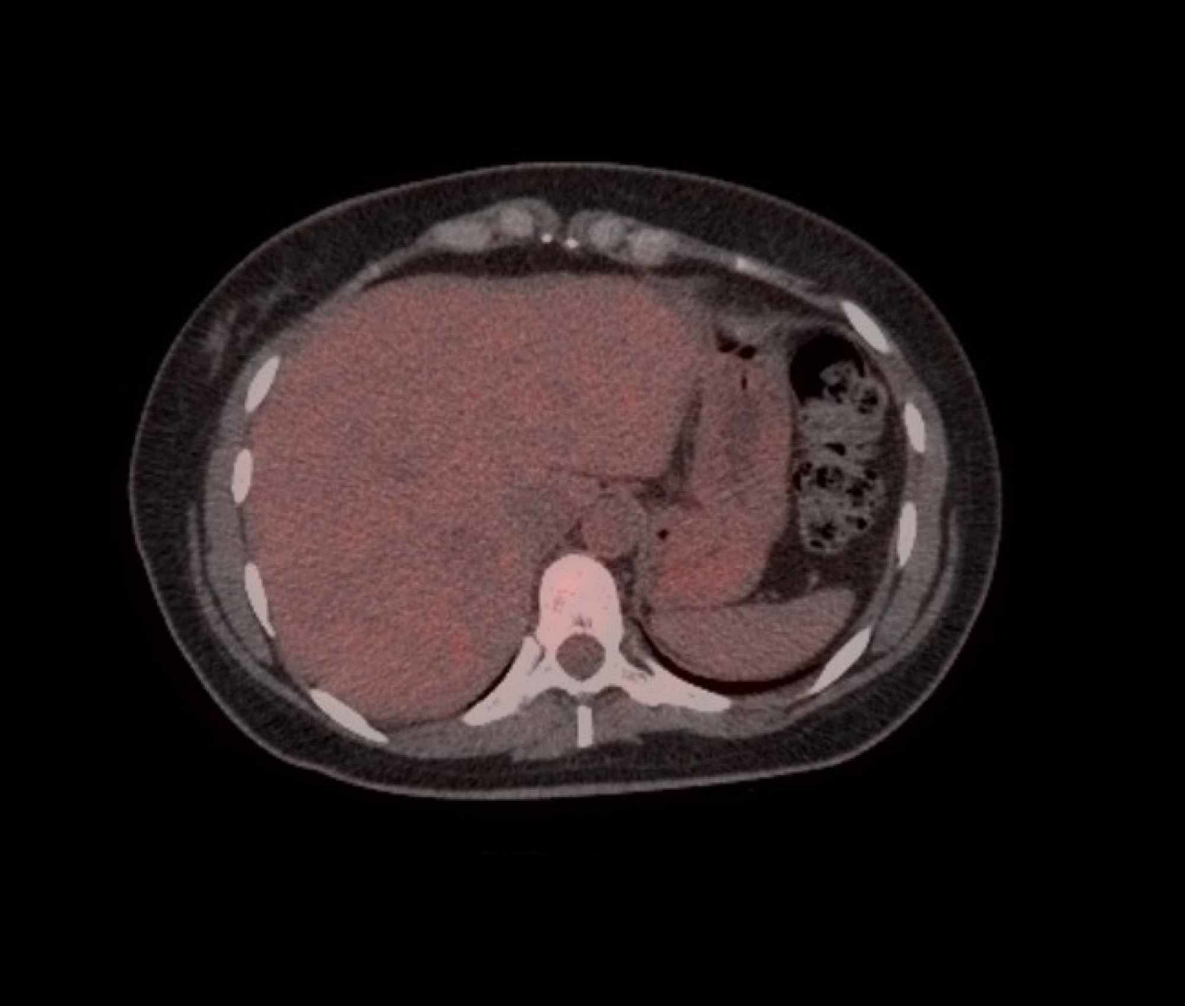
Positron emission tomography (PET) scan done six months prior to admission showing no metastatic foci in the liver

At this point, we revisited history to inquire about the possibility of toxin or drug-induced hepatitis and viral hepatitis. She did not mention any recent addition of new medications. There is no recent acetaminophen intake or any other hepatotoxic medication. She does not consume any alcohol nor has she any history suggestive of a risk for viral hepatitis. Further workup included an ultrasound of the liver, viral hepatitis panel, and toxicology screening. Her viral hepatitis panel and the toxicology screening came back negative with normal serum acetaminophen levels. Ultrasound scan of the liver showed slight intrahepatic dilatation but normal common bile duct and no features of cholelithiasis. There was a 2.7-cm mass in the posterior segment of the right hepatic lobe and the same was confirmed in the CT scan (Figure 1). Of note, there was hepatopetal blood flow seen during ultrasonography (USG)-Doppler and there were no features of portal vein thrombosis. The positron emission tomography (PET) scan which was done at this time revealed multiple new hypermetabolic metastatic lesions in the liver predominantly in the posterior right hepatic lobe (Figure 2). There was increased hypermetabolic activity in the region of the vaginal cuff along with metastatic nodules in the left abdominal wall, left paracolic gutter and right para-aortic region. The previous PET scan done six months ago did not reveal any of the above-mentioned lesions (Figure 3). Over the course in the hospital, she was given supportive and symptomatic treatment.

Her symptoms resolved over the next couple of days. Her liver enzymes remained stable during the hospital stay with a slight downward trend and over the course of next few weeks, she was restarted on chemotherapy with gemcitabine and carboplatin. Two months later while on chemotherapy she developed septic shock and expired.

## Discussion

Liver is a common site for metastasis in patients with malignancies [3]. Both solid tumors and hematological malignancies metastasize to the liver. This case describes a not so common presentation of secondary liver metastasis. There have been a handful of cases in literature with secondary liver metastatic lesions presenting as liver failure. To our knowledge, in literature, there has been no report of a case of cervical cancer with liver metastasis presenting as acute hepatitis.

Kaira et al. in their paper listed 21 cases of acute hepatic failure secondary to hepatic infiltration of metastatic small cell lung cancer. In the case that they reported there was a good response to chemotherapy with improvement in the clinical status and liver function panel [4]. Shakir et al. reported a case of fulminant hepatic failure in a patient with metastatic prostate cancer [5]. Depending on the severity of malignant infiltration obstructing the hepatic vasculature patient can present with features of hepatic ischemic injury, necrosis, and hepatic failure. Cytokine-mediated injury has also been implied in causing hepatic injury in metastatic cancer [6]. The pattern of the liver enzyme elevation is interesting in this case as it mimics acute viral/toxic hepatitis in addition to other causes of marked enzyme elevation as portal or hepatic vein thrombosis and shock liver. Our case is also unique in that the patient did present as an acute liver failure, instead presented as hepatitis with elevated liver enzymes, but no coagulopathy or encephalopathy.

## Conclusions

In patients with history of malignancy, either solid tumors or hematological, if they present with features of acute hepatitis or liver failure, metastatic infiltration of the liver should be high in the differential.
